# Use of synthetic images for training a deep learning model for weed detection and biomass estimation in cotton

**DOI:** 10.1038/s41598-022-23399-z

**Published:** 2022-11-15

**Authors:** Bishwa B. Sapkota, Sorin Popescu, Nithya Rajan, Ramon G. Leon, Chris Reberg-Horton, Steven Mirsky, Muthukumar V. Bagavathiannan

**Affiliations:** 1 Department of Soil and Crop Sciences, Texas A&M University, College Station, TX 77843 USA; 2 Department of Ecosystem Science and Management, Texas A&M University, College Station, TX 77843 USA; 3 Department of Crop and Soil Sciences, North Carolina State University, Raleigh, NC 27695 USA; 4 Sustainable Agricultural Systems Laboratory, USDA-ARS, Beltsville, MD 20705 USA

**Keywords:** Plant sciences, Mathematics and computing

## Abstract

Site-specific treatment of weeds in agricultural landscapes has been gaining importance in recent years due to economic savings and minimal impact on the environment. Different detection methods have been developed and tested for precision weed management systems, but recent developments in neural networks have offered great prospects. However, a major limitation with the neural network models is the requirement of high volumes of data for training. The current study aims at exploring an alternative approach to the use of real images to address this issue. In this study, synthetic images were generated with various strategies using plant instances clipped from UAV-borne real images. In addition, the Generative Adversarial Networks (GAN) technique was used to generate fake plant instances which were used in generating synthetic images. These images were used to train a powerful convolutional neural network (CNN) known as "Mask R-CNN" for weed detection and segmentation in a transfer learning mode. The study was conducted on morningglories (MG) and grass weeds (Grass) infested in cotton. The biomass for individual weeds was also collected in the field for biomass modeling using detection and segmentation results derived from model inference. Results showed a comparable performance between the real plant-based synthetic image (mean average precision for mask-mAP_m_: 0.60; mean average precision for bounding box-mAP_b_: 0.64) and real image datasets (mAP_m_: 0.80; mAP_b_: 0.81). However, the mixed dataset (real image  + real plant instance-based synthetic image dataset) resulted in no performance gain for segmentation mask whereas a very small performance gain for bounding box (mAP_m_: 0.80; mAP_b_: 0.83). Around 40–50 plant instances were sufficient for generating synthetic images that resulted in optimal performance. Row orientation of cotton in the synthetic images was beneficial compared to random-orientation. Synthetic images generated with automatically-clipped plant instances performed similarly to the ones generated with manually-clipped instances. Generative Adversarial Networks-derived fake plant instances-based synthetic images did not perform as effectively as real plant instance-based synthetic images. The canopy mask area predicted weed biomass better than bounding box area with R^2^ values of 0.66 and 0.46 for MG and Grass, respectively. The findings of this study offer valuable insights for guiding future endeavors oriented towards using synthetic images for weed detection and segmentation, and biomass estimation in row crops.

## Introduction

Weeds cause severe crop yield loss, and therefore timely and effective management is key to increasing agricultural productivity. Conventional approaches relying on complete coverage of the field may provide effective weed control but are often inefficient and expensive. Precision weed management can improve resource use efficiency and economics by reducing input use to only those areas where they are needed. This approach, however, can be complex as it comprises several components, including weed detection and actuation systems. The weed detection system is important since it guides the actuation systems. Several detection models have been designed and tested so far^1,2^, but the convolutional neural network (CNN)-based models have revolutionized this domain^3^.

CNN is a class of artificial neural networks that creates numerous feature maps from input images to learn the semantic pattern of the object^4^. Their ability to self-optimize the parameters to improve the pattern recognition process allows for complex object detection. CNNs are increasingly used for weed detection, owing to their high accuracy and less human supervision requirements. Due to its promising nature, researchers have come up with various frameworks designed for their detection needs. For example, Khoshboresh-Masouleh and Akhoondzadeh^5^ developed a lightweight, end-to-end trainable guided feature-based deep learning method, called DeepMultiFuse for weed segmentation in sugar beet (*Beta vulagris* L.) using multispectral images. The existing CNN efforts for weed detection highlight great scope of deep learning frameworks.

CNNs usually require a large number of training samples to obtain high detection accuracy. Manual data annotations for large training sample sets can be tedious and laborious. Additionally, it is at times difficult to obtain large image datasets specific to the needs. Various weed datasets have been made public to date such as “WeedMap”^6^, “DeepWeeds”^7^, “The Crop/Weed Field Image”^8^, etc. These datasets can be very useful in supplementing training data for model development given the similarity in weed species, image resolution and quality between the datasets and training data. However, benefits of these datasets compromise when the targeted environment deviates greatly, and therefore an uniquely customized weed detection model should be developed. One way to deal with these problems would be to create real-looking artificial images that would fit the user's needs and requirements.

An increasingly common approach to image synthesis is to clip the real plants in the imagery, apply modifications to clipped plants, and finally paste over the background images to create synthetic images^9,10^. Few studies have already embraced this concept for weed detection tasks. For example, Gao et al.^11^ created 2271 synthetic images using this approach to train the YOLOv3 and tiny YOLO models in combination with 452 real field images. They applied three types of modifications (zoom, flip, and rotation) to the automatically clipped individual instances and pasted them at a random position within the image. Hu et al.^12^ also used the same approach to create a synthetic image dataset to train weed detection models. They used manually-clipped instead of automatically-clipped plants in the image synthesis procedure. Skovsen et al.^13^ also implemented the same concept to generate large amounts of synthetic images for grass-clover mixtures. While automating the plant clipping process can speed up image synthesis, it is unknown whether it can yield the same levels of accuracy as manually-clipped plants. It is also unknown whether crop positions in the synthetic images affect the performance of detection and segmentation. In addition, how the image synthesis concept works for training an instance segmentation model for weed detection purpose has not been evaluated so far.

It can at times be difficult to obtain real plant images and therefore, fake plants can be great alternatives to real plant instances for generating synthetic images. One of the promising techniques for creating fake images of objects of interest available today is Generative Adversarial Networks (GANs). GANs have been successfully used for various image processing and computer vision tasks ranging from fashion design to video games. Recently, GANs have been utilized for various predictive tasks in the agricultural domain, including weed detection/classification in digital images^14–16^. Espejo-Garcia et al.^14^ tested several architectures and configurations of GANs for creating artificial images of tomatoes and black nightshade. The models trained with these images were able to distinguish between these two plant species with very high accuracy. For example, Wang et al.^17^ used Wasserstein GANs for enhancing RGB images to train semantic segmentation models that could perform pixel-wise segmentation. Very few studies have utilized GANs for weed detection and segmentation and therefore, more investigations are required to assess the full prospects and potential.

In addition to weed localization, biomass information can also be useful for precision weed control systems. With this information, herbicide spray systems can be configured to deliver spray output optimized for plant biomass, thus saving resources. Studies have used various techniques for estimating plant biomass using digital technologies. For example, Harkel et al.^18^ used LiDAR-derived 3D point clouds to estimate biomass for winter wheat, potato, and sugar beet. Using the depth and RGB image-based volume reconstruction method, Andújar et al.^19^ calculated 3D mesh volume for weeds to estimate weed biomass. Although these 3D techniques are proven to result in more accurate biomass estimation, they are computationally more expensive and often inefficient^20^. A simple 2D approach that utilizes plant coverage information has been studied by Skovsen et al.^13^. However, these studies have investigated biomass estimation in a unit area, rather than that of individual plants, which is critical for precision management. The current study explores the feasibility of a simple 2D approach coupled with instance segmentation for estimating biomass at the individual plant level.

In this study, the main goal was to investigate various methods of generating synthetic images for training a Mask R-CNN model for weed detection and segmentation. The objectives of the study were:

1. Explore the potential of synthetic images for training and building a Mask R-CNN-based weed detection and segmentation model.
  - Evaluate the effect of crop row arrangement, instance diversity, and clipping method on synthetic image quality and training performance.
  - Compare the training performance of real plant-based vs fake plant-based synthetic images.
  - Evaluate performance gain with the mixed dataset (real + synthetic images).
2. Assess the potential of a Mask R-CNN model-based segmentation results in estimating the above-ground biomass of weeds.

## Methods

### Study area and experimental setup

The study was conducted in the summers of 2020 and 2021 at the Texas A&M AgriLife Research farm (30° 32′ 15″ N, 96° 25′ 35″ W; elevation: 70 m). The location is characterized by a sub-tropical climate, with an average monthly maximum and minimum temperature during the study period (May–June) of 32.3 °C and 21.3 °C, respectively. Cotton was chosen as the model row crop for this study. Glyphosate-resistant (Roundup Ready^®^) cotton was planted at the seeding rate of 100,000 per hectare on May 1, 2020 and April 20, 2021, respectively, using a 4-row seed drill (row spacing: 1 m). Cotton was grown following the recommended production practices for the region. The dominant weed species in the study area were a mix of morningglories (*Ipomoea* spp.) that comprised of tall morningglory [*Ipomoea purpurea* (L.) Roth.] and ivyleaf morningglory (*Ipomoea hederacea* Jacq.), Texas millet [*Urochloa texana* (Buckley) R.D. Webster], and johnsongrass [*Sorghum halepense* (L.) Pers.]. Some other weed species occurred at low frequencies, including Palmer amaranth (*Amaranthus palmeri* S. Watson), prostrate spurge (*Euphorbia humistrata* Engelm.), and browntop panicum (*Panicum fasiculatum* Sw.). At the time of image collection, weed species occurred at different growth stages, from cotyledon to about five true leaves (Table 1). Experimental research and field studies on plants complied with institutional, national, and international guidelines.

**Table 1:** Crop-weed conditions during real image dataset acquisition in 2020 and 2021 using a Fujifilm camera.

Image dataset name	Acquisition date	Cotton growth stage	Weed composition/growth stage	Weed density (plants m^−2^)
Cotton1	May 06, 2020	4–5 leaves	MG: cotyledon-4 leaves JG: 2–3 leaves TM: 2–3 leaves	18
Cotton2	June 13, 2021	2–4 leaves	MG: cotyledon-6 leaves TM: 2–4 leaves	21

*MG* morningglories, *JG* johnsongrass, *TM* Texas millet.

### Data collection

#### High-resolution digital images

A 100-megapixel FUJIFILM GFX100 medium format mirrorless RGB imaging camera was integrated with a multi-copter drone, Hylio AG-110 (Hylio Inc., TX, USA) to capture high-resolution aerial images of the cotton in summer of 2020 and 2021, hereafter referred as Cotton 1 and Cotton 2 dataset, respectively. The images were captured by the drone operating at 4.9 m above ground level and at a speed of 0.61 m/s. The FUJIFILM GF 32–64 mm f/4 R LM WR lens was set at a focal length of 64 mm, shutter speed at 1/4000 s, ISO at 1250, and f-stop at 8, which resulted in high-quality images with a spatial resolution of 0.0274 mm/pixel at the flying height specified above. All the images were stored in standard PNG format at 16-bit depth. Cotton 1 was the main dataset and comprised 560 images, out of which 460 were reserved for the training and validation dataset, and the remaining 100 as hold-out test dataset. Whereas, Cotton 2 had 100 images for test purposes.

#### Above-ground weed biomass collection

A total of 15 quadrats (1 m^2^) were randomly placed in a cotton field (0.12 ha) for weed biomass collection in 2020 and 2021. Each plant in the quadrat was clipped at the ground level and stored in separate paper bags. The location of each weed in the quadrat was physically mapped on a paper for later use during image analysis to identify and reference each individual. In total, 60 morningglories (MG) and 60 grass (Grass) weed instances were clipped in 2020, and 39 and 44, respectively in 2021. The clipped plants were dried in an oven at 60 °C for 48 h for dry biomass measurement. The plants were clipped from the experimental area complying all the Texas A&M AgriLife protocols.

### Methodology for objective 1

**Figure 1:**
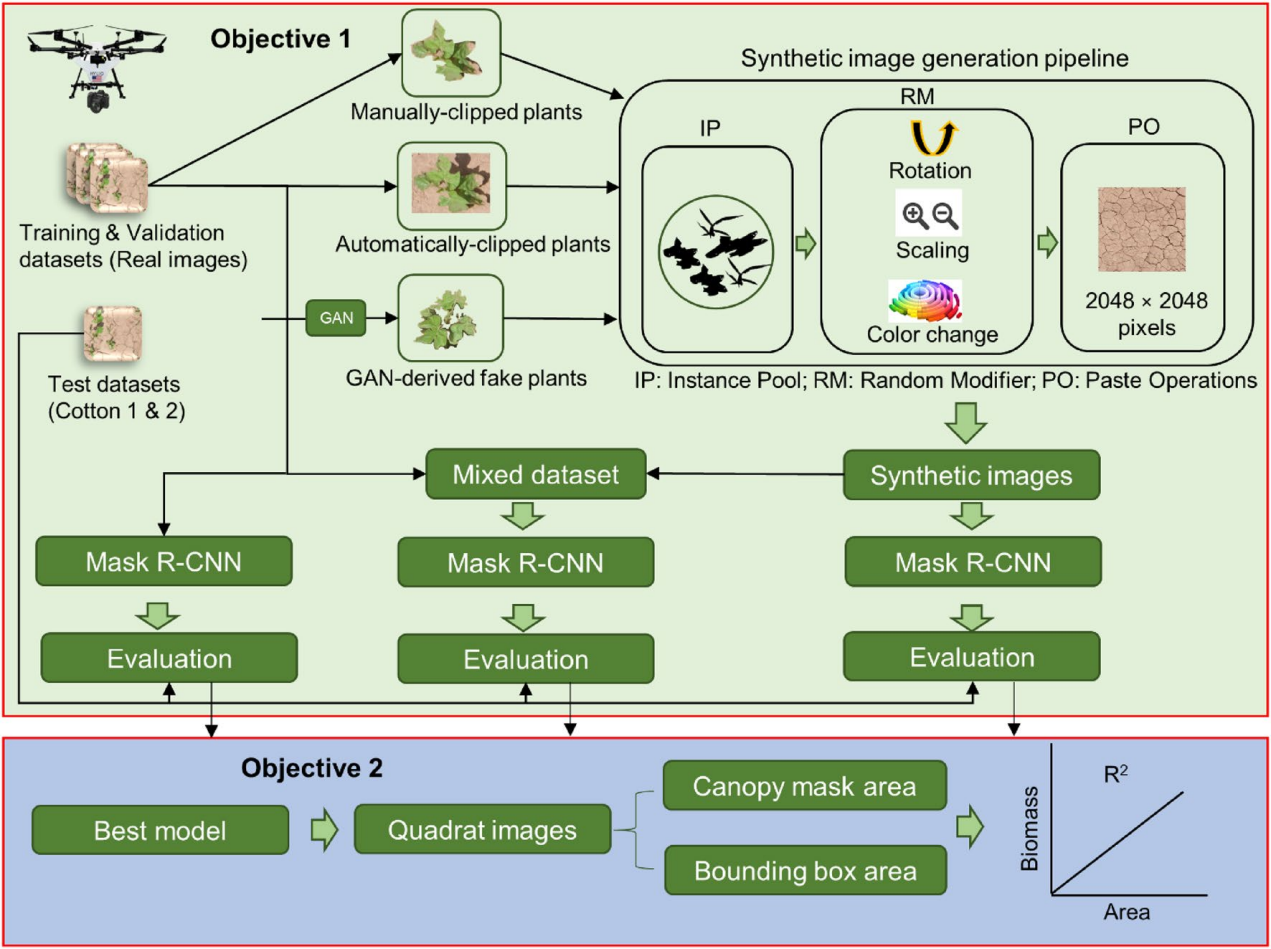
Workflow diagram for the methodology implemented in this study. The pale green and blue sections show the schematic for objectives 1 and 2, respectively. Objective 1 aimed at testing several models with different-source input images, whereas the objective 2 determined the predictability of model results to estimate above-ground biomass of weeds.

The general workflow for this experiment is shown in Fig. 1. The workflow shows the progression of major methodological steps undertaken for both objectives.

#### Synthetic image generation pipeline

The synthetic image generation pipeline (SIGP) consisted of three main components (Fig. 1). The first component included the instance pool (IP), which consisted of individual plants clipped either from real images or individual fake plants generated and stored in a 4-band (RBGA) PNG format. The second component included a random modifier (RM) algorithm that randomly obtained instances from the IP and applied several modifications to these instances. The modifications were made in three ways: (a) rotating instances by a random angle between 0 and180°, (b) transforming instances with a random size factor ranging between 0.6 and 1.2, and (c) changing digital values for hue and saturation of instances by 0–10%. The third component included a paste operator (PO) algorithm that pasted modified instances at user-defined or random locations in the soil background images to create synthetic images. Five representative soil background images of 2048 × 2048 pixels were clipped from real images acquired using the Fujifilm camera. The PO recorded information on the locations where instances were pasted as well as several other metadata such as instance id, instance category, image id, etc. to create an annotation dictionary for each image. The PO finally merged the annotation directories for all the images to create an annotation file in the *.json* file format. The pipeline was programmed in such a way that each image would have 4 instances for each of the three categories: (a) cotton, (b) MG, and (c) Grass. The algorithm logic used in SIGP is provided in Algorithm 1.

**Figure:**
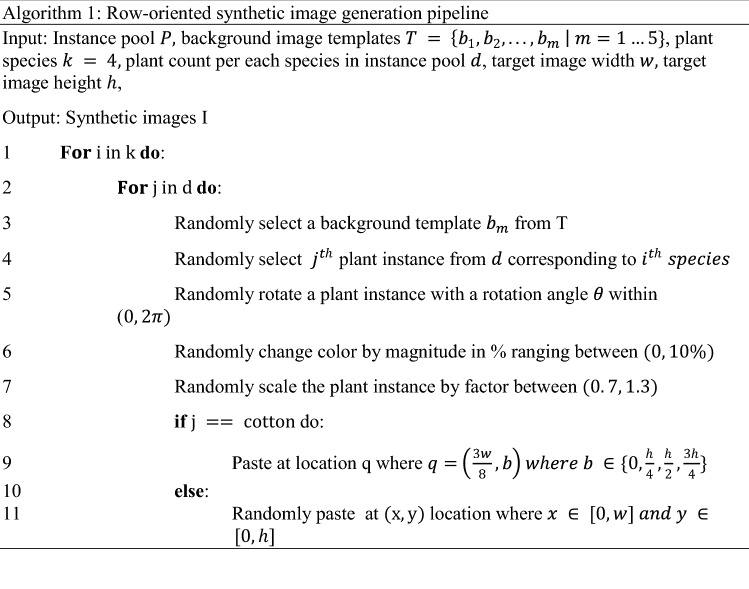

#### Effect of crop row arrangement

**Figure 2:**
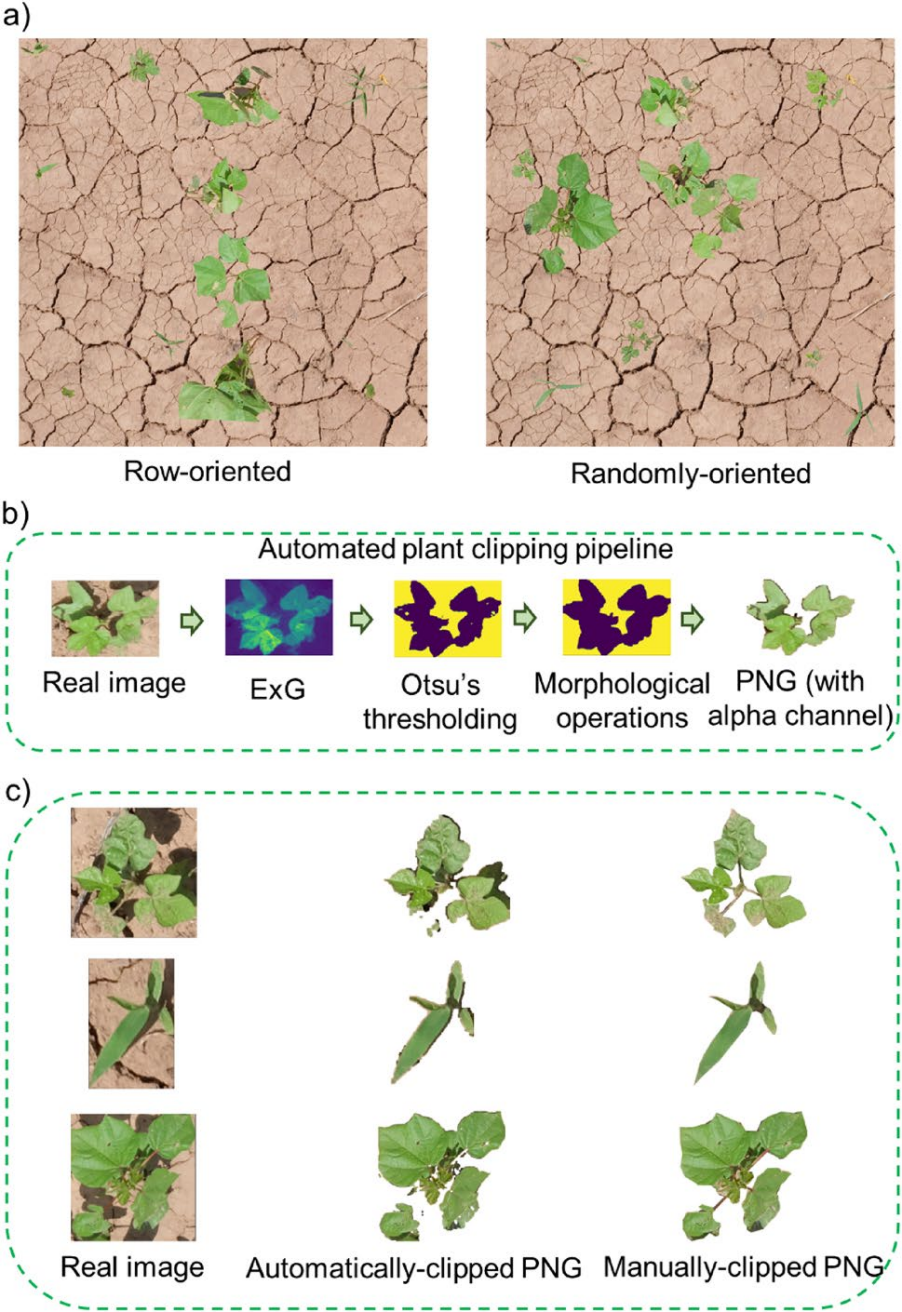
(**a**) A representative sample for row-oriented and randomly-oriented images produced with the synthetic image generation pipeline, (**b**) The automated plant clipping pipeline to derive PNG images with alpha channel, and (**c**) Comparison of automatically-clipped and manually-clipped PNG instances for the given real plant images.

The aim was to evaluate if the row arrangement of crops was important for row crops such as cotton in the synthetic images for better training results. To test this, two sets of synthetic images were generated: (a) images with cotton lined-up in a row (row-oriented), and (b) images with cotton pasted in random locations (randomly-pasted) (Fig. 2a). First, real plants for cotton, MG, and Grass were clipped to canopy boundary from the real images to create an IP. Fifty instances were clipped for each class. Second, the SIGP was implemented with a slight change in the PO, which was programmed to paste the cotton instances coming from RM in two ways: (a) following user-defined locations to line up in a row for row-oriented images, and (b) following machine-generated random locations for random-oriented images. The synthetic image sets were then used for training the model separately. In order to evaluate how the results change with image resolution, another set of synthetic images with reduced image resolution (512 × 512 pixels) for both arrangements was generated. Assessments were made to compare the performance of the models.

#### Effect of instance diversity

The aim was to determine how the IP size influences the performance of the Mask R-CNN model. It was hypothesized that the more the number of unique plant instances used in SIGP, the more the variance captured by the synthetic images, and thus better the training. Altogether, seven IPs with varying sizes were created that contained 1, 5, 10, 20, 30, 40, and 50 instances from each class. Seven different synthetic image and annotation datasets were created from respective IPs, which were then used to train and build detection models. Individual assessments were made to compare the performance of each model.

#### Effect of instance clipping method

The goal was to compare the performance of the models trained with synthetic images generated using plant instances clipped manually and automatically. The real images captured with the Fujifilm camera were subjected to the automated plant clip pipeline (APCP) (Fig. 2b). The algorithm logic used in APCP is shown in Algorithm 2. First, the excess greenness index (ExG) was calculated for selected real images using Eq. (1). Second, Otsu’s method was employed to mask the bareground. Finally, an alpha channel was added to the resultant image to create a 4-band (RBGA) PNG image. The plant instances clipped automatically or manually (Fig. 2c) were fed into SIGP to generate individual sets of synthetic images.

1 $$ExG = \frac{2\times g - r - b}{r+g+b},$$

where $g, r, \mathrm {and} \,b$ represent digital values for green, red, and blue channels, respectively.


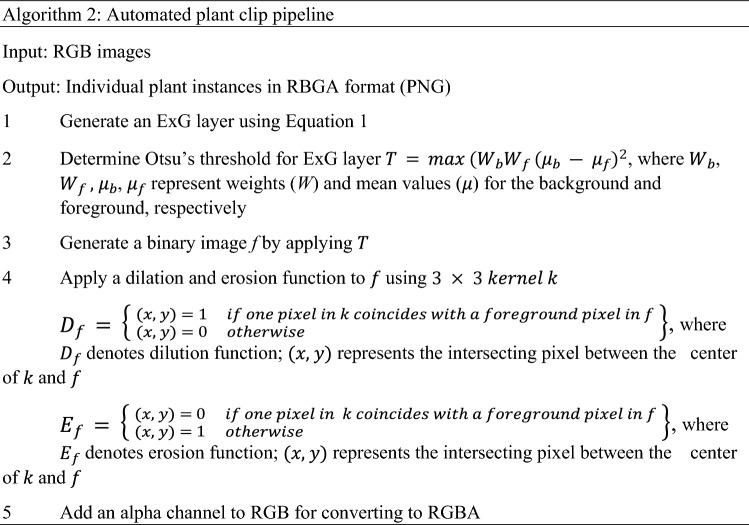


#### Performance of real plant instances vs. fake plant instances

**Figure 3:**
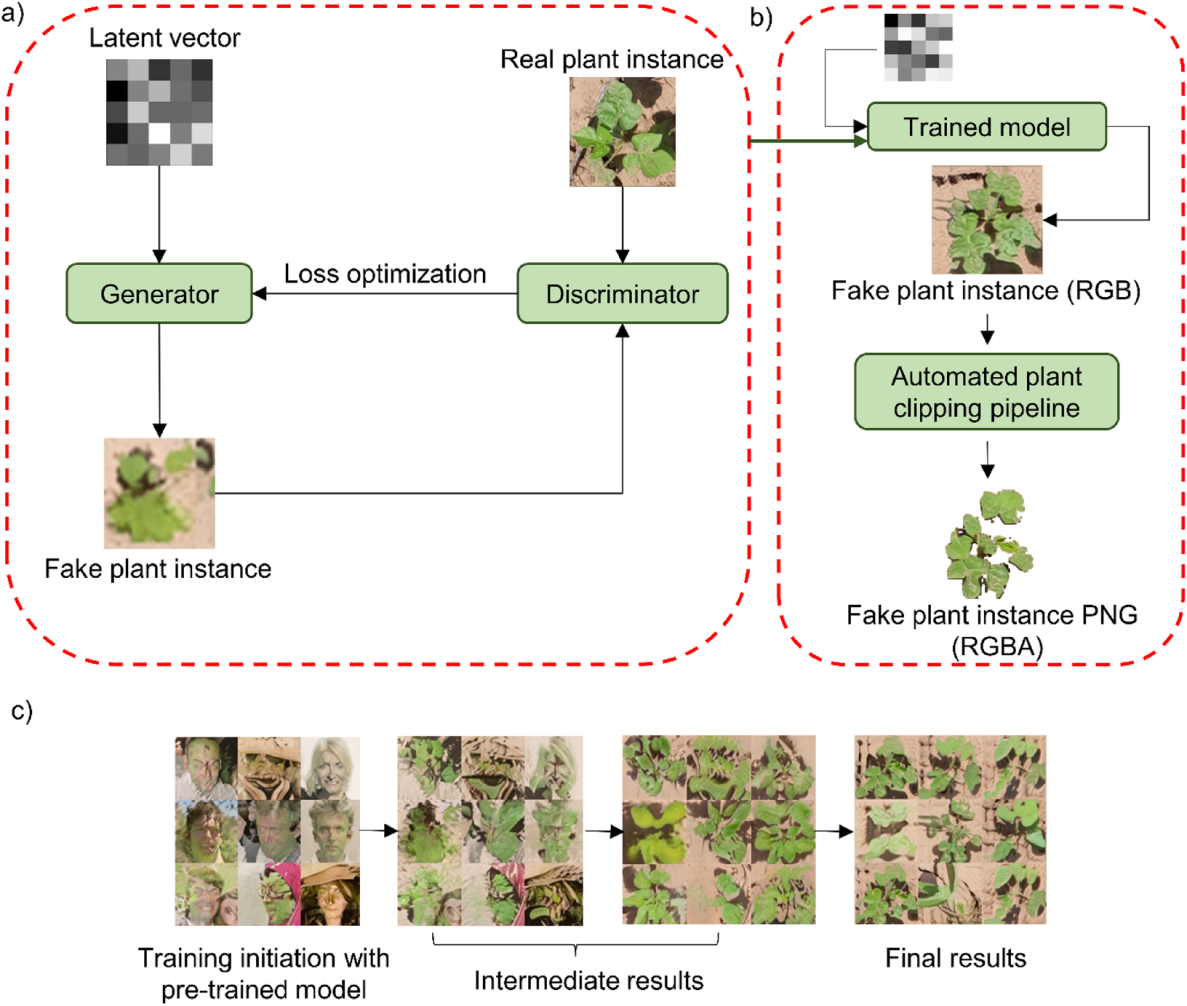
(**a**) Schematic showing the general workflow for a simple generative adversarial network (GAN) model, (**b**) Additional post-processing step for generating new fake plant PNGs using the custom trained styleGAN model, and (**c**) sample results obtained with the custom styleGAN model at various stages of the training process. The faces shown in first sub-figure are fake and do not exist, and are provided by styleGAN-ada repository. The styleGAN-ada script used this fake face template for training initation during the model training process.


The aim was to compare the performance of fake plant-based synthetic images with real plant-based synthetic images. An improved GAN framework called StyleGAN2 with adaptive discriminator augmentation (StyleGAN-ADA) developed by NVIDIA^21^ was used in this study to generate fake plants. GANs are essentially composed of two main networks, a generator and a discriminator (Fig. 3a). The generator deterministically generates samples from latent variables, whereas the discriminator distinguishes samples from the real dataset and the generator. The model was trained with 50 instances of each class using the official TensorFlow implementation code provided in (https://github.com/NVlabs/stylegan2-ada). The training samples were subjected to an on-the-fly augmentation process to increase the sample size. A pre-trained network model ‘ffhq256’ was used as the base model for transfer learning. After model training, approximately 50 fake plant instances in 3-band format were generated for each class. The “trunc” and “seeds” parameter were set to 1 and 1–500, respectively while generating images. The generated images were then passed through the APCP method to create a new IP comprised of 4-band images in PNG format (Fig. 3b). The new IP was subjected to SIGP to create a unique set of synthetic images. The sample results obtained at different training phases of GAN is shown in Fig. 3c. The algorithm logic for the GAN process is provided in Algorithm 3.

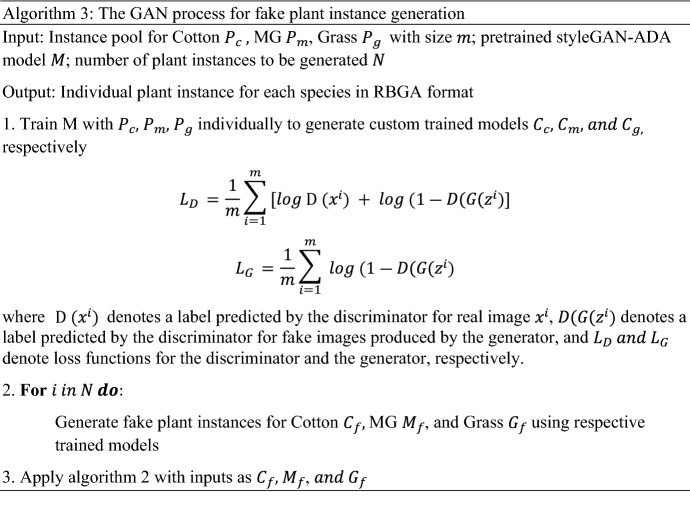


#### Performance gain with mixed dataset

The idea was to compare the performance of a mixed dataset (real images + synthetic images) with just a synthetic image-derived dataset. For this, a total of 460 real images acquired using the Fujifilm camera sensor were manually annotated for Cotton, MG, and Grass individuals. The polygon annotations were drawn for each plant in all the images. The synthetic image dataset was combined with a real image dataset to create the mixed dataset. The synthetic dataset that yielded the highest accuracy in the earlier analysis was chosen for inclusion in the mixed dataset. The mixed dataset comprised a total of 1230 images and 18,352 annotations that were used to train the Mask R-CNN model.

#### Model training and accuracy assessment

Mask R-CNN, an instance segmentation model^22^, was used for weed detection and segmentation in this study. Mask R-CNN is similar to its predecessor “Faster R-CNN” framework, except that it has an additional mask branch that results in an object mask in addition to the bounding box. Both Faster R-CNN and Mask R-CNN are two-stage object detectors composed of two modules. The first one is a Region Proposal Network (RPN) that proposes several object candidate regions in the image using anchors. The second module is a detector that works in two steps. First, it extracts features from dense feature maps for the regions selected during RPN and in the second step, it calculates the confidence score for each region that contains the object of interest^22^. Detectron2-a PyTorch-based modular object detection library^23^ written in Python language (https://github.com/facebookresearch/detectron2), was used to implement Mask R-CNN in this study. A pre-trained model provided by the repository was used for transfer learning. Mask R-CNN was trained, validated, and tested with different sets of images generated/acquired in real-world settings to evaluate the effects as discussed earlier in the manuscript (Table 2). The configurations set for the model are provided in Table 3.

**Table 2:** Details on training, validation, and test datasets used in this study.

Image dataset	# of images	# of annotations	Annotation composition
Real_training_image_dataset	460	9115	Cotton: 7.65% MG: 17.8% Grass: 75.01%
Synthetic_training_image_dataset	770	9237	Cotton: 33.34% MG: 33.32% Grass: 33.34%
Real_validation_image dataset	100	995	Cotton: 7.65% MG: 17.8% Grass: 75.01%
Real_test_image_dataset (Cotton1)	100	848	Cotton: 12.66% MG: 45.31% Grass: 42.01%
Real_test_image_dataset (Cotton2)	50	976	Cotton: 10.04% MG: 7.4% Grass: 82.01%

**Table 3:** Major hyperparameters and values used with Mask R-CNN training.

Major hyperparameters	Values
BACKBONE	ResNet101
EPOCH*	50,000
BASE_LEARNING_RATE^a^	0.001
LEARNING_RATE_SCHEDULER_NAME	WarmupMultiStepLR
MOMENTUM	0.9
WEIGHT DECAY	0.0001
RPN_BATCH_SIZE_PER_IMAGE^a^	256
RPN_NMS_THRESHOLD^a^	0.7
ANCHOR_SIZES	[32, 64, 128, 256, 512]
NUMBER OF CLASSES*	3
CHECKPOINT_PERIOD*	5000
TEST_EVAL_PERIOD*	1000

Hyperparameters with * were used with custom values. Hyperparameters with ^a^ were tuned by testing a set of values before selecting the best value. The rest of the hyperparameters were used with the default values. The description of the hyperparameters can be found at https://detectron2.readthedocs.io/en/latest/modules/index.html.

**Figure 4:**
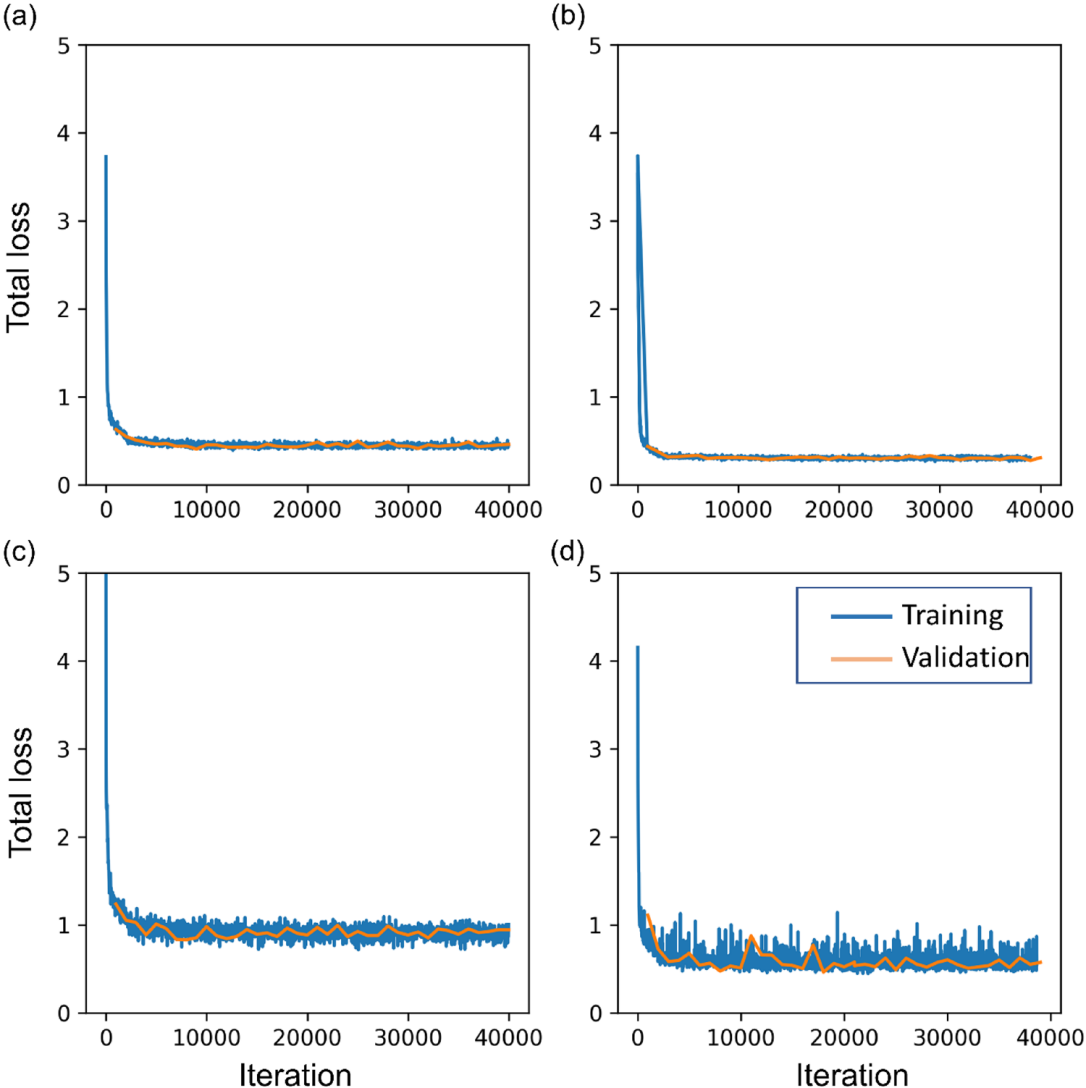
Graph showing a total loss for training and validation across iterations for various models, including the model trained with (**a**) real plant instance-based synthetic images, (**b**) GAN-derived plant instance-based synthetic images, (**c**) real images, and (**d**) a mixed dataset of real and synthetic images.

The training process took 5–6 h, depending on model objectives and structure. Since the input image size to the model was 2048 × 2048, the IMGS PER GPU parameter was set to 1 because setting value more than 1 resulted in “CUDA OUT OF MEMORY” error. Resizing an image to lower the dimension before feeding into the model was also not an option since doing so would decrease the ability to visualize very small grass in the images. Due to longer training time, it was not possible to tune all the parameters. Therefore only few parameters were selected based on prior experience and literature and were tuned by testing a set of values. It should be noted that the annotation proportion of the real image training dataset was imbalanced between the species. To minimize the bias associated with an imbalanced dataset in training, a data sampler known as “RepeatFactorTrainingSampler” was used instead of a regular data sampler while training the model. This sampler first computes per-image repeat factors based on category frequency for the rarest category for the given image and creates a list of image indices that need to be repeated while feeding into the model in each epoch (https://detectron2.readthedocs.io/en/latest/_modules/detectron2/data/samplers/distributed_sampler.html). The training and validation loss across the iterations during the training of the most accurate model using real, synthetic, and mixed datasets is given in Fig. 4.

Cotton1 and Cotton2 test datasets were used to assess all the models trained in this study. The standard performance metric called Mean Average Precision (mAP) was calculated to assess the performance of the Mask R-CNN model. mAP was calculated separately for both model results, bounding box (bbox) and mask. mAP for these results is hereafter referred as mAP_b_ and mAP_m_, respectively. In recent years, these metrics have been frequently used to assess the accuracy of object detection and segmentation tasks^11,12^. mAP is a mean of AP calculated for each class to be detected/predicted by the model. AP for each class is calculated as the area under a precision-recall curve. The area is determined in two stages. First, the recall values are evenly segmented to 11 parts starting from 0 to 1. Second, the maximum precision value is measured at each level of recall and averaged to determine AP (Eq. 2).

2 $$\mathrm{AP }= \frac{1}{11}\sum_{r \in \{0, 0.1, 0.2...1\}}{p}_{max }\left(r\right),$$

where $${p}_{max}$$ represents maximum precision measured at respective recall ($$r)$$ level.

Precision and recall values are in turn calculated using Eqs. (3) and (4), respectively.

3 $$\mathrm{Precision }= \frac{TP}{TP +FP},$$

4 $$\mathrm{Recall }= \frac{TP}{TP +FN},$$

where $TP$, $FP$, and $FN$ denote true positive, false positive, and false-negative samples, respectively.

True positives, false positives, and false negatives are identified using the Intersection over Union (IoU) ratio criterion. This ratio is calculated by comparing the ground truth box/mask with the model predicted box/mask. The predicted box/mask is labeled as TP if the ratio is above the user-defined threshold. In this study, the threshold for IoU was set to 0.5. The mAP value ranges between 0 and 1, with 0 indicating null accuracy and 1 representing perfect accuracy.

### Methodology for objective 2

The second objective of the study was aimed at assessing the potential of detection results (i.e. weed canopy masks and bounding boxes) in estimating above-ground weed biomass. The best model evaluated among all the models developed earlier was chosen for this purpose. The model was applied to the test images that contained the weeds sampled for biomass measurements. Both the detected bounding box and segmented canopy mask area of respective weeds were calculated and regressed separately with above-ground biomass collected for each species. The coefficient of determination was calculated to assess the biomass predictability of model outputs.

## Results and discussion

### Effect of crop row arrangement

**Figure 5:**
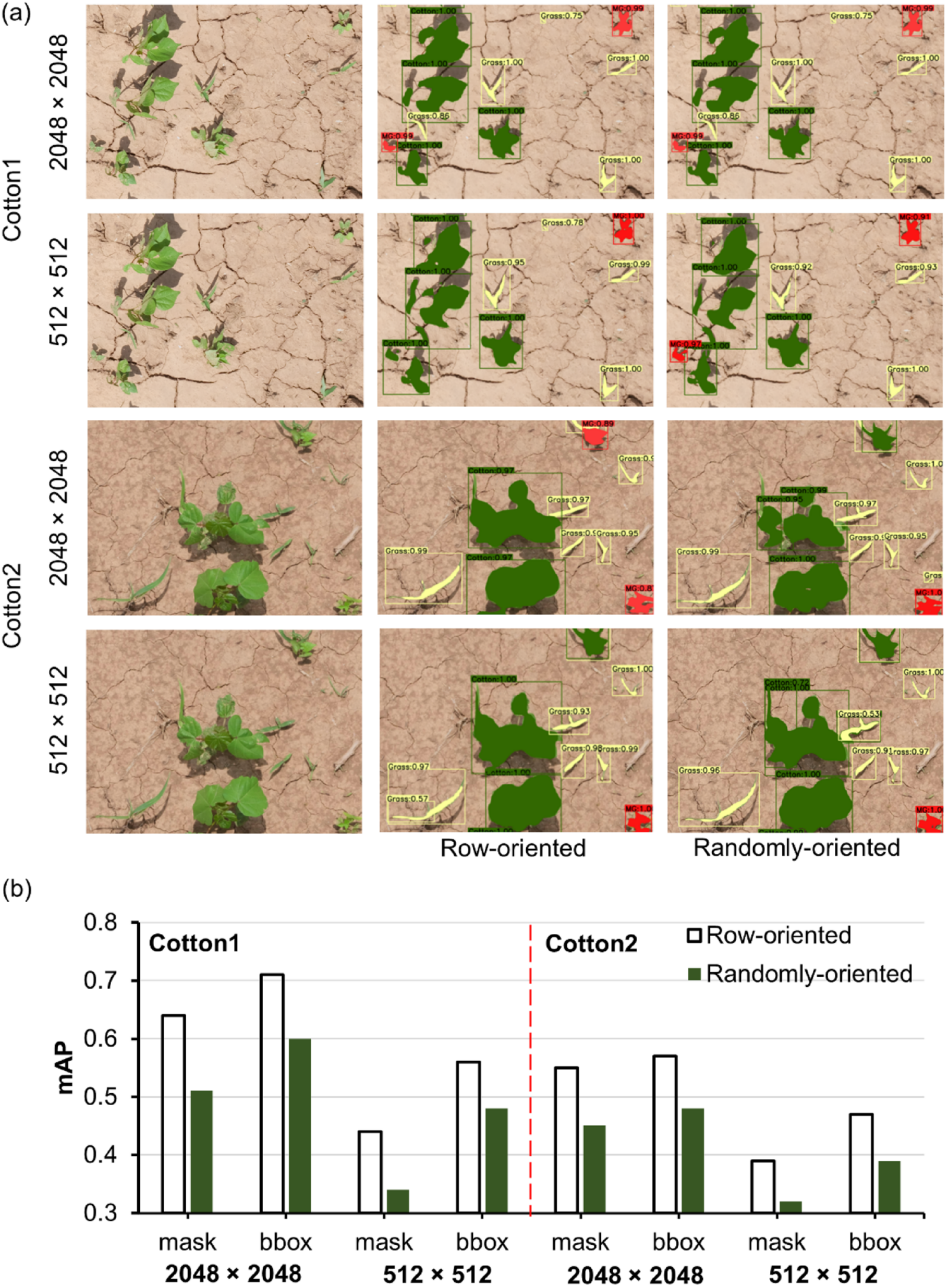
Results obtained from models trained with row-oriented and randomly-oriented synthetic images: (**a**) Detection and segmentation results obtained for both test datasets (Cotton1 and Cotton2) with the original (2048 × 2048) and reduced image size (512 × 512), and (**b**) mAP values (mask and bbox) obtained for Cotton1 and Cotton2. Here, green, red, and yellow detected masks represent cotton, morningglories, and grass, respectively.

Two different sets of synthetic images were produced (row-oriented and randomly-oriented) to test the importance of crop row orientation in the images for model performance. The row-oriented dataset resulted in higher mAP_b_ and mAP_m_ for both cotton datasets compared to the randomly-oriented dataset. This was true when training the model at both the original size (2048 × 2048 pixels) and at a reduced resolution (512 × 512 pixels) (Fig. 5). The accuracy was greater when training was performed on the original size images. The detection and segmentation results using both sets of images are shown in Fig. 5. The discrepancy in performance was more obvious for Cotton2 than for Cotton1. The Mask R-CNN framework used the ResNet101 backbone for feature extraction. This backbone has 101 layers that can learn many patterns and complex features at various scales^24^. It is likely that the row-arrangement of cotton was well-recognized by the edge detector filters at the shallow layers of ResNet, which may have contributed to efficient learning at higher levels. The higher-level feature map may have highlighted cotton row as a prominent feature as these maps are derived from a series of convolution operations from lower-level feature maps.

### Effect of instance diversity

**Figure 6:**
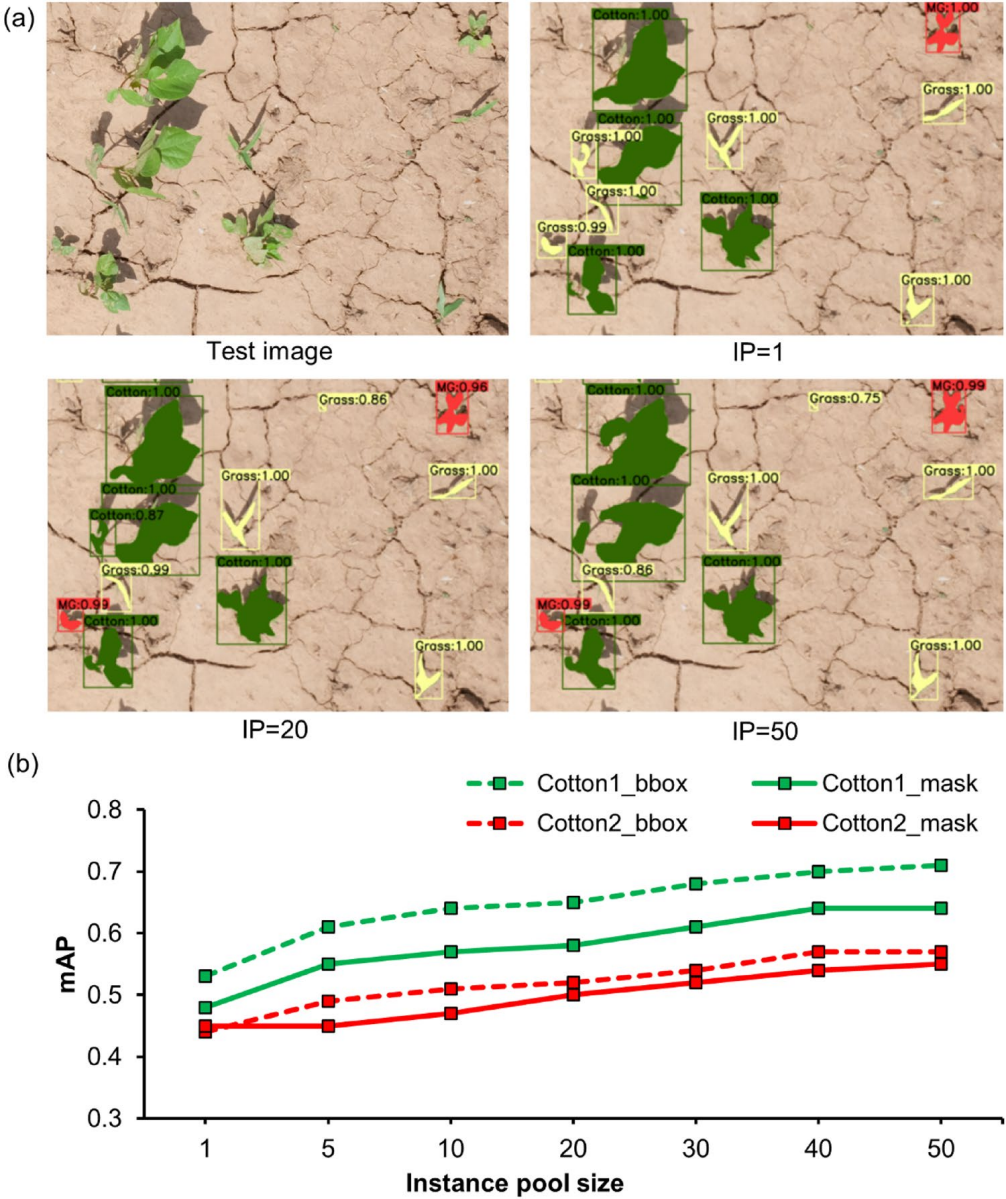
Results obtained from models trained with synthetic images generated using various instance pool (IP) sizes: (**a**) Detection and segmentation results obtained for Cotton1 with IP = 1, IP = 20, and IP = 50, and (**b**) Mean average precision (mAP) values compared for bounding box (bbox) and mask results for Cotton1 and Cotton2, obtained for IP sizes ranging from 1 to 50. Here, green, red, and yellow detected masks represent cotton, morningglories, and grass, respectively.

Seven different sets of synthetic images were generated with different IP sizes. The main goal was to evaluate the effect of IP size on the synthetic image quality and performance. As expected, the performance differed across different IP sizes (Fig. 6a). In general, the mAP_b_ and mAP_m_ showed an increasing trend with increases in IP from 1 to 50 for both the cotton datasets (Fig. 6b). It is notable that an IP size of 1 resulted in a satisfactory average mAP_b_ and mAP_m_ value of 0.49 and 0.47, respectively. The rate of increase flattened towards an IP size of 40, indicating that any further increase in IP size may not necessarily improve the performance significantly. Hu et al.^12^ also observed no significant improvements beyond an IP size of 68 when training a Faster R-CNN model with synthetic images. Overall, results indicate that quality synthetic images can be generated even with low IP sizes, given that the samples are truly representative of the objects of interest.

### Effect of clipping methods

**Figure 7:**
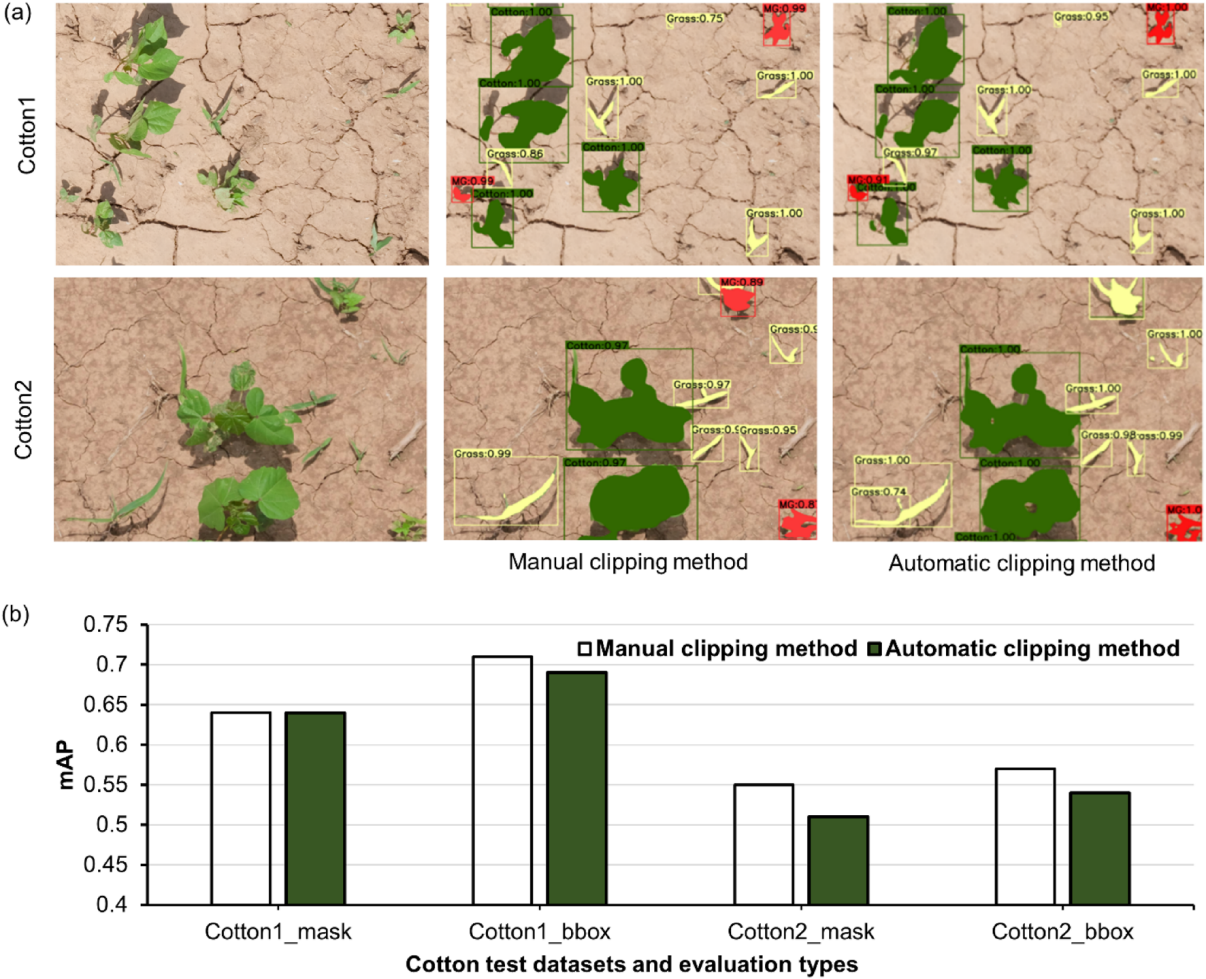
Results obtained with models trained with synthetic images generated using manual clip and automatic clip method: (**a**) Detection and segmentation results obtained for both test datasets (Cotton1 and Cotton2), and (**b**) Mean average precision (mAP) values for bounding box (bbox) and mask results obtained for Cotton1 and Cotton2. Here, green, red, and yellow detected masks represent cotton, morningglories, and grass, respectively.

Models were trained using two different sets of synthetic images generated with the IP that comprised of manually-clipped and automatically-clipped plants. The overall results for Cotton1 and Cotton2 showed that automatically-clipped plants can perform comparably to the manual-clipping method (Fig. 7). In particular, mAP_b_ and mAP_m_ were similar between the two clipping methods for both cotton datasets. In total, it took approximately 170 min to manually clip 150 plant instances, whereas the same instances were clipped and sorted automatically just in 5 min, at a 34-fold faster rate. Gao et al.^11^ also successfully used automatically clipped plant instances to generate synthetic images for training a weed detection model. The present study evaluates these two common methods by testing for a multi-class detection and mask generation problem.

### Performance of GAN-derived fake plants

**Figure 8:**
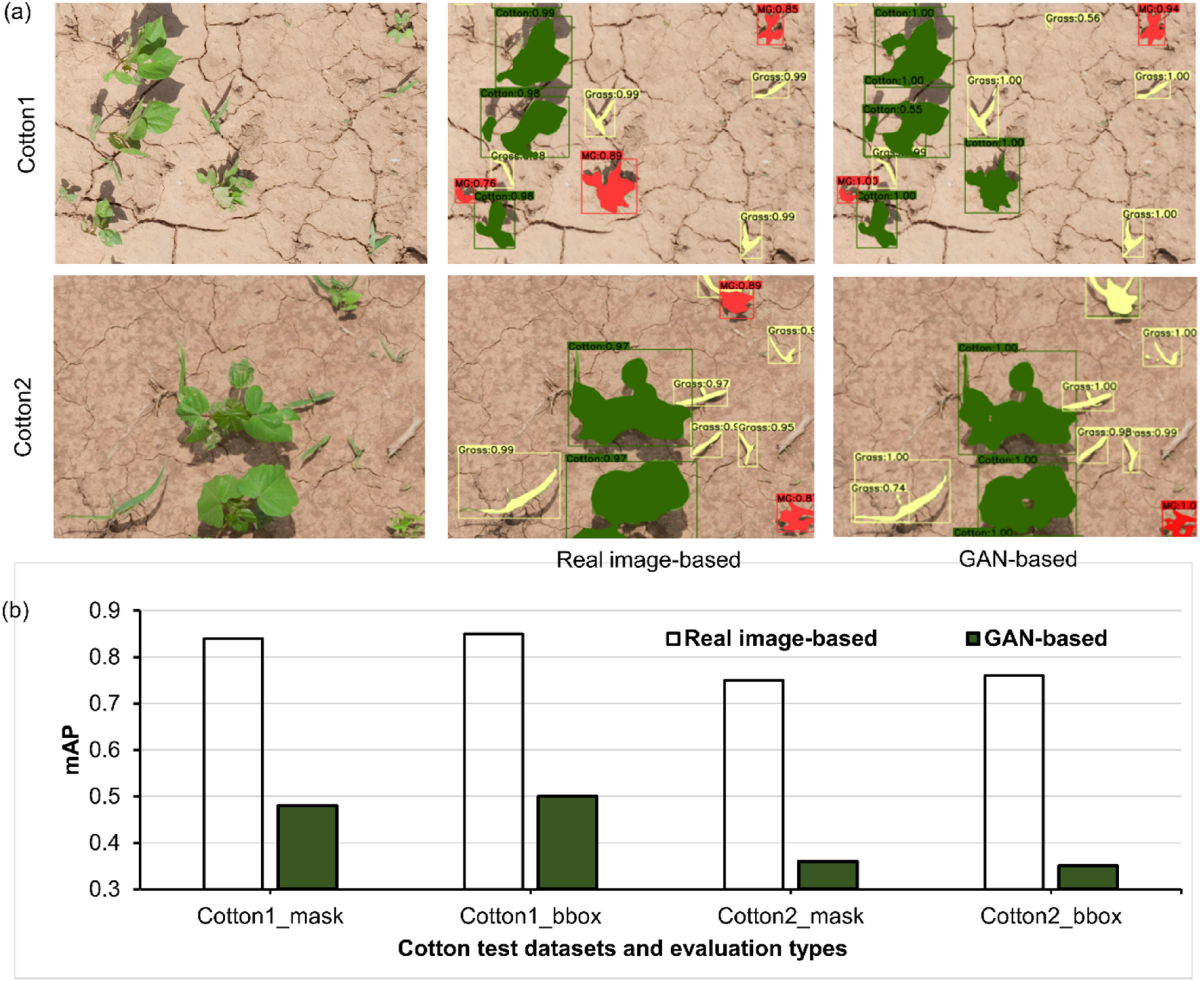
Results obtained from models trained with synthetic images generated using real plant instances and generative adversarial network (GAN)-derived fake plants: (**a**) Detection and segmentation results obtained for the test datasets Cotton1 and Cotton2, and (**b**) Mean average precision (mAP) values for bounding box (bbox) and mask results obtained for Cotton1 and Cotton2. Here, green, red, and yellow detected masks represent cotton, morningglories, and grass, respectively.

**Figure 9:**
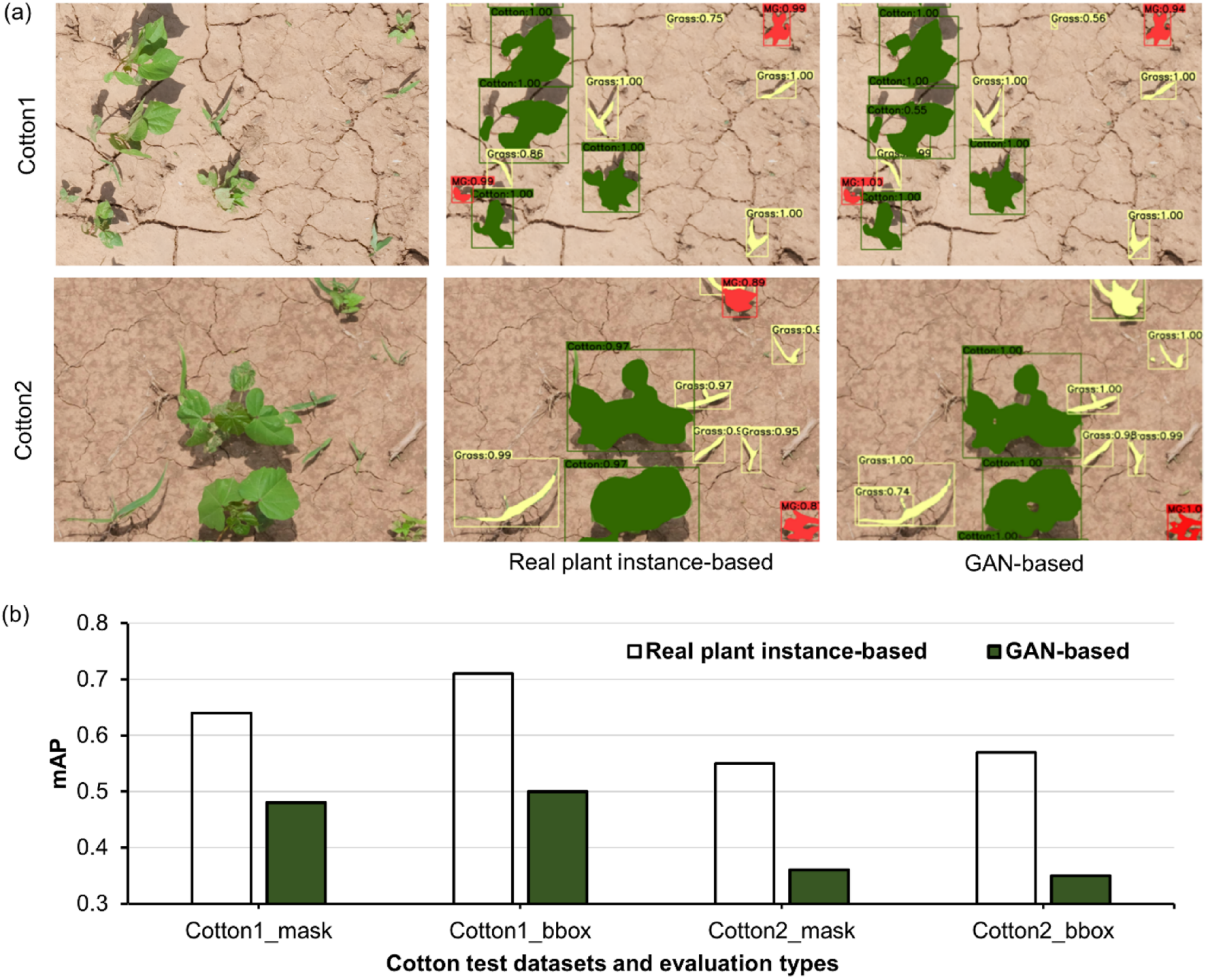
Results obtained from models trained with synthetic images generated using real plant instances and generative adversarial network (GAN)-derived fake plants: (**a**) Detection and segmentation results obtained for the test datasets Cotton1 and Cotton2, and (**b**) Mean average precision (mAP) values for bounding box (bbox) and mask results obtained for Cotton1 and Cotton2. Here, green, red, and yellow detected masks represent cotton, morningglories, and grass, respectively.

The performance of GAN-derived fake plant-based synthetic images and real plant-based synthetic images was evaluated to independently train the Mask R-CNN model. Both the real images (Fig. 8) and real plant-derived synthetic images (Fig. 9) resulted in a greater accuracy compared to GAN-derived fake plant-based synthetic images. In general, MG was misclassified as cotton and vice-versa, whereas such errors were less common with Grass. High similarity in leaf appearance between cotton and MG could be attributed to misclassification between them. It is likely that the training sample size employed for styleGAN in our case (~ 50 plants per class) was not sufficient for generating high-quality fake plants. However, our results show that the GAN approach can be a promising technique for training the weed detection and segmentation model. The potential of this technique may increase with an increase in training data size. Fawakherji et al.^15^ utilized a conditional GAN to generate realistic multispectral synthetic images and used them in combination with the original images in the training process. They observed improvements in the segmentation performance by the model.

### Performance of the mixed dataset vs. real dataset

**Figure 10:**
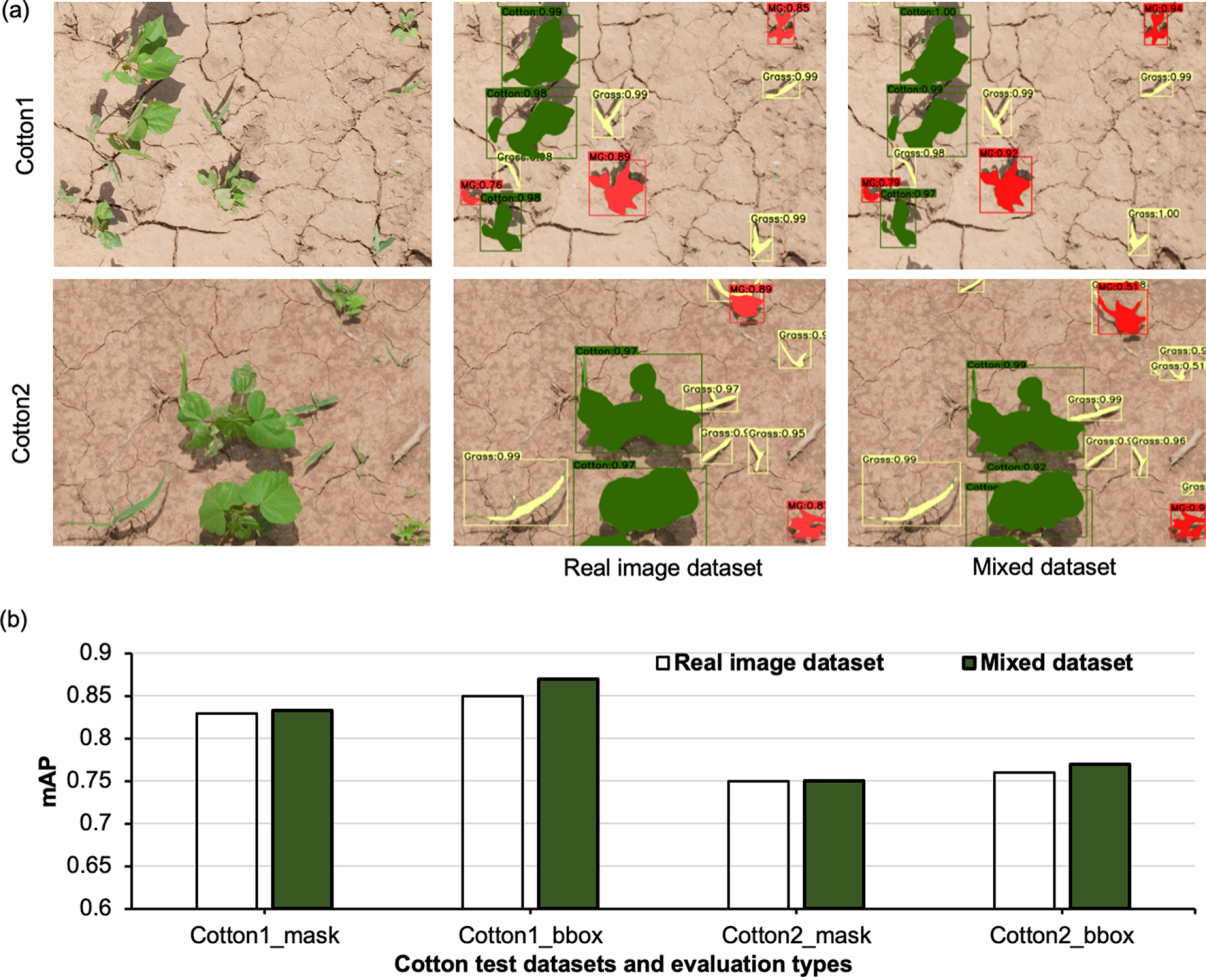
Results obtained from models trained with real image dataset and mixed dataset (original real images + real plant-based synthetic images): (**a**) Detection and segmentation results obtained for the test datasets Cotton1 and Cotton2, and (**b**) Mean average precision (mAP) values for bounding box (bbox) and mask results obtained for Cotton1 and Cotton2. Here, green, red, and yellow detected masks represent cotton, morningglories, and grass, respectively.

The mixed dataset did not result in a significant performance gain in this study. For both cotton datasets, the performance of the mixed dataset was generally comparable to the real dataset (Fig. 10). This finding doesn’t conform to previous reports (e.g. Ref.^11^) that found considerable improvements in accuracy with the addition of synthetic images to real image dataset for training. The lack of improvement in accuracy in our study could be attributed to the fact that the real images utilized here contained sufficient variance for different objects of interest. Additional synthetic images didn’t add much variance to the training samples. In the future, different proportions of real and synthetic images can be tested to identify the critical minimum number of real images required to observe synthetic image synergistic effect.

### Performance analysis in relation to past studies

Several studies in the past have looked into utilizing CNN frameworks to detect weeds in crops. Liu and Bruch^25^ used the You Only Look Once (YOLOv2), a lightweight CNN framework, to detect weeds in romaine lettuce in digital RGB images and achieved the highest mean average precision (mAP) value of 0.91. Salazar-Gomez et al.^26^ achieved an mAP value of 0.875 with YOLOv5 in detecting weeds in sugarbeet. Yu et al.^27^ detected dandelion (*Taraxacum officinale* F.H. Wigg.), ground ivy (*Glechoma hederacea* L.), and spotted spurge (*Euphorbia maculata* L.) in perennial ryegrass (*Lolium perenne* L.) using DetectNet frameworks with an F-score > 0.92. Lottes et al.^28^ employed CNN with an encoder-decoder structure to detect weeds in sugar beet and achieved an average F-score of 0.92. Our study stands comparable to these studies in terms of detection accuracy. In addition, our study evaluated models for mask segmentation as well not only using real images but also synthetic images.

### Assessing the biomass predictability of model outputs

**Figure 11:**
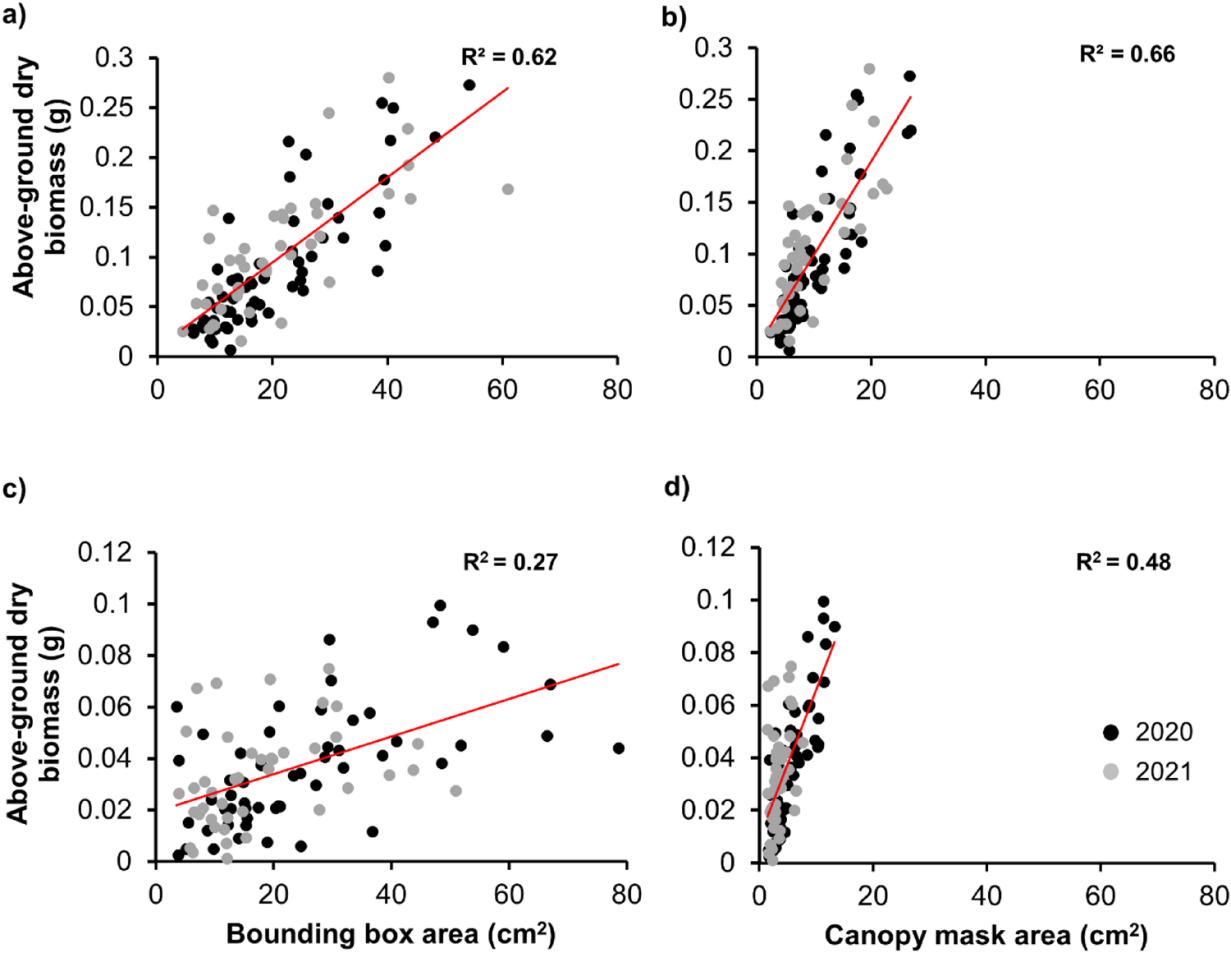
Regression analysis for estimating biomass for morninglories (**a**,**b**) and grasses (**c**,**d**) with bounding box and canopy mask area, respectively. The red line represents the best fitted line estimated by the regression analysis. Altogether, 99 (60 in 2020 and 39 in 2021) and 104 (60 in 2020 and 44 in 2021) MG and Grass individuals were sampled for biomass, respectively.

The area of the bounding boxes and canopy masks resulting from Mask R-CNN were regressed independently with the biomass of respective weeds to determine the biomass predictability of model outputs. The regression was performed separately for MG and Grass. For both groups, the canopy mask area was found to be a better estimator compared to the bounding box area. Further, biomass was estimated more accurately for MG (R^2^ = 66) than for Grass (R^2^ = 0.48) with canopy mask area (Fig. 11). The bounding boxes overestimate the leaf surface area, which is not systematic owing to the varying canopy structure of plants. This problem is pronounced in the case of Grass due to the random orientation of Grass leaves, resulting in the increased bounding box area. This could be the prime reason why the canopy mask area was more effective in estimating biomass than the bounding box area. The reason for better prediction of MG biomass compared to that of Grass using mask area could be that Grass had extremely low biomass and such biomass measurements were prone to errors. Albert et al.^29^ effectively used canopy coverage area to estimate weed dry biomass in a grass-clover mixture. Skovsen et al.^13^ also found a linear association between model-predicted visual weed fraction in pixels and fractional weed biomass in kg. Both of these studies investigated weed biomass estimation in a unit area that included multiple plants. However, the current study explored the feasibility of biomass prediction at an individual plant level, which is crucial for site-specific weed management. Overall, the results indicate that the canopy mask area can be a reliable estimator of biomass, especially for broadleaved weeds.

## Conclusions

This study explored various strategies for generating synthetic images in training a Mask R-CNN model for weed detection and segmentation. The feasibility of biomass estimation with the Mask R-CNN model outputs was also assessed. The important take-aways from this study are:

- Synthetic images can be a great alternative to real images. In this study, real plant instance-based synthetic images provided ~ 80% of the accuracy that was achieved with original real images.
- Row-orientation of cotton in the synthetic images proved to be beneficial compared to random orientation. This calls for a careful selection of crop positions in the images while generating synthetic images.
- About 40–50 real plant instances were sufficient for generating synthetic images for optimal performance. This implies that the quality (i.e., variability) of plant instances can be prioritized over the number of plant instances.
- Synthetic images generated with automatically-clipped plant instances performed comparably to the ones generated with manual clipping. This suggests that time and other resources could be saved by clipping plant instances automatically.
- The GAN-derived fake plant instance-based synthetic images did not provide comparable accuracy to real plant instance-based synthetic images. However, it should be noted that a small training sample size was used in this study for training the GAN model, which may have resulted in low-quality synthesis.
- Weed segmentation output (i.e., canopy mask area) can be a good estimator for biomass, especially for broadleaved weeds.

The findings presented here do not apply to every situation, and they have some practical limitations. For example, automatic clipping may be challenging under complex crop-weed background scenes, including occlusions. Further, the optimal IP size reported in this study may not be sufficient for other row crops and weed species depending on the level of variability in the population. This study in general advances our understanding of how synthetic images can be exploited to train weed detection models for precision weed management. The scope of this work is more justified in light of the time-demands associated with the model training and precision weed management effort. While this study focused on the use of the models over the static images, the findings can be extrapolated to on-premise real-time weed detection. Future research should investigate how this approach performs with different weed densities and growth stages. More training samples should be used in training GAN to fully harness its potential and improve accuracy.

## Data Availability

That dataset used and/or analyzed during the current study are available from the corresponding author on reasonable request. Authors confirm that the plants were not purchased/gifted from any source.
